# Nuclear survivin is a prognosticator in gastroenteropancreatic neuroendocrine neoplasms: a meta-analysis

**DOI:** 10.1007/s00432-022-04013-1

**Published:** 2022-04-15

**Authors:** Sarah Krieg, Christoph Roderburg, Stephen Fung, Tom Luedde, Wolfram Trudo Knoefel, Andreas Krieg

**Affiliations:** 1 Clinic for Gastroenterology, Hepatology and Infectious Diseases, Heinrich-Heine-University and University Hospital Duesseldorf, Duesseldorf, Germany; 2 Department of Surgery (A), Heinrich-Heine-University and University Hospital Duesseldorf, Moorenstr. 5, Bldg. 12.46, 40225 Duesseldorf, Germany

**Keywords:** Survivin, BIRC5, GEP-NEN, Neuroendocrine neoplasm

## Abstract

**Purpose:**

Gastroenteropancreatic neuroendocrine neosplasms (GEP-NEN) are biologically heterogenous tumors with an increasing incidence over the past decades. Although efforts have been made in the treatment of these tumors, survival rates in metastasized tumor stages remain frustrating. Thus, there is an urgent need to identify novel targets as alternative treatment options. In this regard, the inhibitor of apoptosis protein (IAP) family member survivin could be such an attractive target. Therefore, aim of our meta-analysis was to assess the role of survivin as a biomarker and predictor in GEP-NEN.

**Methods:**

Medline, Web of Science and Scopus were screened for studies that fulfilled our selection criteria. Quality assessement of the studies was based on design, methodology, generalizability and results analysis. Meta-analyses were conducted using a random-effects model and effect size measures were expressed as pooled Hazard Ratio (HR) or Odds Ratio (OR) with 95% Confidence Interval (CI).

**Results:**

Six eligible studies with 649 patients (range 77–132) assessed survivin expression in GEP-NEN by immunohistochemistry. High expression levels of nuclear survivin in GEP-NEN correlated with a shorter overall survival (HR 3.10; 95% CI 2.15–4.47; *p *< 0.0001). In contrast to cytoplasmic survivin (OR 1.24; CI 0.59–2.57; *p *= 0.57), nuclear survivin was also associated (OR 15.23; CI 3.61–64.23; *p *= 0.0002) with G3/poorly differentiated GEP-NEN.

**Conclusion:**

Nuclear Survivin is highly expressed in more aggressive G3 GEP-NEN and correlates with a poor outcome. Survivin is therefore an interesting molecule for a targeted therapy, especially for patients with highly proliferative G3 GEP-NENs.

## Introduction

Gastroenteropancreatic Neuroendocrine Neoplasms (GEP-NENs) represent a heterogeneous group of malignancies that are defined by the expression of the neurosecretory vesicle proteins synaptophysin (SYN) and chromogranin A (CgA) (Rindi et al. 1986; Buffa et al. 1987). According to the 5^th^ edition of the WHO classification, GEP-NEN are now classified based on their morphology and proliferative activity into well differentiated G1 (Ki-67 index ≤ 2%), G2 (Ki-67 index 3–20%) or G3 (Ki-67 index > 20%) neuroendocrine tumors (NETs), poorly differentiated neuroendocrine carcinomas (NECs, Ki-67 index > 20%) and mixed neuroendocrine/non-neuroendocrine neoplasm (MiNEN) (Klimstra et al. 2019). However, recent data demonstrate that GEP-NEN are genomically unrelated tumors. Whereas pancreatic NETs exhibit frequently genetic alterations in MEN1, DAXX, ATRX, MUTYH, CHEK2, BRCA2, and genes involved in the mammalian target of rapamycin (mTOR) signaling pathway (Jiao et al. 2011; Scarpa et al. 2017), NECs more commonly demonstrate mutations in TP53 and RB1 (Yachida et al. 2012; Takizawa et al. 2015). In addition, mutations in BRAF can be found in almost 50% of colorectal NECs (Dizdar et al. 2019).

Based on the Surveillance, Epidemiology, and End Results (SEER) database the incidence of GEP-NEN dramatically increased up to 3.56 per 100.000 persons in the United States between 1973 and 2012 (Dasari et al. 2017). One explanation for the rising incidence could be an improvement in diagnostic imaging methods and the awareness of these tumors. At the same time, however, a more favorable prognosis for patients with metastasized GEP-NEN is observed (Dasari et al. 2017). This observation is possibly due to the advances in the interdisciplinary therapy of GEP-NEN. Whereas complete surgical resection remains the first-line therapy for patients presenting with a localized disease, advanced tumor stages require interdisciplinary treatment concepts such as metastasectomy, chemotherapy, targeted therapies, interventional procedures, or peptide receptor radionuclide therapy (PRRT).

However, survival for patients with metastasized disease remains poor with a median overall survival of 12 months when compared to localized or regional disease stages (Dasari et al. 2017). It is therefore important to identify attractive molecular targets in GEP-NEN that are accessible to targeted therapy. Recently, we demonstrated in GEP-NEC in vitro as well as in vivo that inhibitor of apoptosis protein (IAP) family member survivin/BIRC5 could be such a druggable target (Dizdar et al. 2017). Both, a knock down by shRNAs as well as the transcriptional repression of survivin by small molecule antagonist YM155 demonstrated a pronounced effect on cell viability and tumor formation in a xenograft mouse model (Dizdar et al. 2017).

Survivin, composed of 142 amino acids, is the smallest member of the IAP-family, containing only a single Baculovirus IAP Repeat (BIR) domain at the N-terminus and a C-terminal *α*-helix (Wheatley and Altieri 2019). Via the *α*-helix, survivin interacts with borealin and inner centromer protein (INCENP) to regulate the activity of aurora-B-kinase within the chromosomal passenger complex (CPC), implicating its role in the coordination of chromosome segregation, cytokinesis and mitosis (Wheatley and Altieri 2019). While nuclear survivin is involved in the regulation of cell division, cytoplasmic survivin orchestrates intracellular pathways during programmed cell death and tumor cell invasion. Because survivin does not directly bind to caspases, a family of cysteine proteases which are well characterized effectors and executioners of apoptotic cell death, indirect mechanisms such as the stabilization of caspase-inhibitor X-linked inhibitor of apoptosis protein (XIAP) or an interaction with hepatitis B X-interacting protein (HBXIP) have been proposed to explain survivin actions during apoptosis (Marusawa et al. 2003; Dohi et al. 2004). Importantly, recent data demonstrate the relevance of survivin in mediating drug resistance (Park et al. 2011). In this context, survivin protects endothelial cells of the tumor-supplying vessels from cell death caused by chemotherapeutic agents (Tran et al. 2002).

Beyond this role, a survivin-XIAP complex seems to facilitate metastasis by inducing tumor cell invasion via fibronection-mediated activation of cell mobility kinases (Mehrotra et al. 2010). These experimental data are supported by meta-analyses and immunohistochemical studies that have shown an association between high survivin expression and blood vessel invasion as well as lymph node or distant metastasis i.e. in malignant tumors of the thyroid gland (Werner et al. 2016), lung (Fung et al. 2021), colon (Krieg et al. 2013b) and stomach (Krieg et al. 2013a).

To date, only a small number of studies have focused on the prognostic value of survivin and its role as a biomarker in GEP-NEN. Our goal was therefore to identify those publications in a systematic review to synthesize their data in a meta-analysis using the Population, Intervention, Comparison, Outcome (PICO) model (Richardson et al. 1995) to clarify the question if in patients with GEP-NEN (P) survivin expression (I) correlates with clinicopathological variables and poor outcome (CO).

## Methods

### Literature search

Our systematic review with meta-analysis was carried out in accordance with the AMSTAR (Shea et al. 2007) and PRISMA (Moher et al. 2009) checklist. First, we performed an electronic literature search using Medline, Web of Science and Scopus that was updated on December 27^th^, 2021 to identify those articles that focused on the expression of survivin in GEP-NEN. Therefore, we combined keywords such as “Neuroendocrine”, “tumo*”, “carcinoma”, “neoplas* “NEN”, “NET”, “NEC”, “survivin” and “BIRC5” by Boolean operators. There was no restriction for language and publication year applied.

### Selection criteria

Study selection was performed according to the following in- and exclusion criteria: (1) expression of survivin was quantified by immunohistochemistry (IHC), fluorescence in situ hybridization (FISH) or reverse transcription and polymerase chain reaction (RT-PCR) analysis; (2) tissue specimen from patients with GEP-NEN were used for the detection of survivin; (3) an association between survivin expression levels and survival or clinicopathological parameters was investigated; (4) Hazard ratios (HR) with Confidence interval (CI) were extractable from survival analysis; (5) clinicopathological variables with respect to survivin expression were extractable to calculate the Odds Ratio (OR); (6) in case of dual publication the more detailed study was included; (7) studies that analyzed survivin expression in other biological materials then tissue specimens (i.e. blood cells, serum, plasma, urine) were excluded as well as studies presenting data from The Cancer Genome Atlas (TCGA).

### Data extraction

First, two investigators (S.K. and A.K.) independently reviewed all abstracts obtained from the database search to select those articles that potentially investigated the expression of survivin in tissue specimen of GEP-NEN. Next, full texts from these abstracts were rigorously screened and if eligble included in our meta-analysis. Therefore, both investigators separately extracted and integrated the data in a database by including the first author’s name, year of publication, country of origin, number of patients, follow-up, clinicopathological information, source of tissue samples, laboratory methodology including the detection method and cut-off values, number of events and total number of patients with respect to the investigated variable, as well as Hazard Ratio (HR) with confidence interval (CI). Finally, both investigators compared the entire datasets.

### Quality assessment

Quality assessment was based on the score which has been proposed by the European Lung Cancer Working Party (ELCWP) (Steels et al. 2001). Briefly, this score consists of four different categories, scientific design, laboratory methodology, generalizability and results analysis, whereby each category can be rated with a maximum of 10 points. Thus, in total, a maximum of 40 points is achievable. Accordingly, higher scores reflect a better study quality. Two investigators (S.K. and A.K.) calculated for each included study the scores and discussed these results to reach a consensus if necessary.

### Statistical analysis

The HR served as effect size measure to analyze an association between survivin expression and survival. A HR > 1 indicated that high survivin expression predicted a poor outcome. In studies in which the HR was not provided by the authors, but a Kaplan–Meier survival curve, the data were extracted directly from the survival curves using the Engauge Digitizer software version 12.1 (http://digitizer.sourceforge.net/). We then reproduced the Kaplan–Meier curves (GraphPad Software, Inc, La Jolla, CA, USA), assuming that the censored events remained constant over the follow-up period and estimated the HR with its 95% CI using logrank test.

Odds Ratios (ORs) provided information about an association between the expression of survivin and clinicopathological variables. For this purpose, the number of cases with positive expression in the specific group of the analyzed clinicopathological parameter was set in relation to the total number of examined cases in the group. Statistical heterogeneity was tested using the Cochrane’s Q test (Chi-squared test; Chi^2^), with a Chi^2^ higher than the degree of freedom (*df*) and a low *p* value (*p *< 0.1) indicating heterogeneity. In addition, inconsistency (*I*^2^) statistic quantified heterogeneity (Higgins and Thompson 2002). Whenever heterogeneity became evident, one-way sensitivity analysis was performed. Since we had to assume that each study estimated a different effect, we used a random-effects model. Effect size measures were expressed as pooled OR or HR with 95% CIs according to the method of DerSimonian and Laird and data were presented as forest plot (Paule and Mandel 1982). Funnel plots were drawn and visually inspected for asymmetry as an indicator for publication bias. Egger’s (Egger et al. 1997) and Begg and Mazumdar (1994) test were performed to statistically assess the risk of publication bias. For binary outcomes, the risk of publication bias was tested as recently proposed (Harbord et al. 2006; Peters et al. 2006). Meta-analysis was performed using R version 4.1.1 and the meta package.

## Results

### Study characteristics and quality

**Fig. 1:**
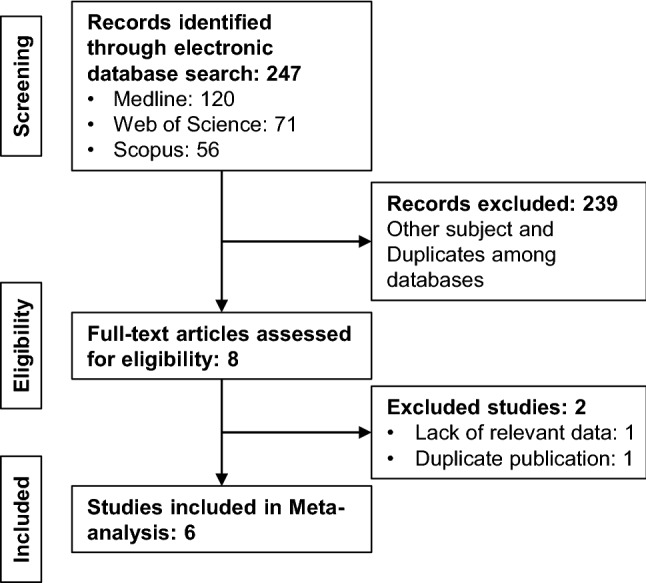
Flow diagram summarizing the process of electronical database screening and study selection

Based on our search strategy, we were able to identify 120, 56 and 71 potentially relevant articles in the Medline, Scopus and Web of Science, respectively (Fig. 1). After careful reading of the abstracts, we included 8 articles and meticulously screened the full-texts (Grabowski et al. 2005; Drozdov et al. 2009; Ekeblad et al. 2012; Cherenfant et al. 2014; Fotouhi et al. 2016; Dizdar et al. 2017; Briest et al. 2018; Hanif et al. 2020). During this step, 1 study (Briest et al. 2018) was excluded because it was a duplicate to the study by Grabowski et al. (2005) and 1 study did not provide survival or clinicopathological data (Drozdov et al. 2009). Finally, 6 studies published between 2005 and 2020 and originating from 4 different countries with a total of 649 patients (range 77–132) were considered eligible for our meta-analysis (Grabowski et al. 2005; Ekeblad et al. 2012; Cherenfant et al. 2014; Fotouhi et al. 2016; Dizdar et al. 2017; Hanif et al. 2020). These included 331 women and 318 men, most (*n* = 582) of whom received surgical therapy for G1-3 tumors. Of note, only 1 study (Grabowski et al. 2005) provided information regarding adjuvant treatment strategies and 1 study also included 62 lung NETs (Hanif et al. 2020). Table 1 summarizes further characteristics of the included studies. All studies examined the association between expression levels of survivin and overall survival, 2 studies also provided data on progression-free survival (Table 2). In addition, 5 studies investigated a potential association between survivin and clinicopathological parameters in GEP-NEN. The majority of studies classified GEP-NEN according to the WHO classification 2000, one study (Dizdar et al. 2017) used the updated version of 2010. Technically, the expression of survivin was evaluated immunhistochemically in all studies on formalin-fixed and paraffin-embedded (FFPE) tissue sections. While one study did not describe the antibody used in detail, the remaining studies detected survivin by different clones. In addition, cut-off values that defined a positive or high expression of survivin varied among the studies. Interestingly, all studies, with the exception of the study by Hanif and colleagues (Hanif et al. 2020), which only analyzed nuclear survivin, described the most frequent expression of survivin in the cytoplasm. To determine the quality of the included studies we took advantage of the recently published ECLWP score, which incorporates the quality of study design, laboratory methodology, generalizability and results analysis. This ultimately resulted in the global quality score, which we expressed as a percentage of the maximum achievable score for each study (Table 3).

**Table 1:** Clinical characteristics of included studies

First author	Year	Country	Study period	Sex (female/male)	No. of patients	Age (median)	G	Therapy/source of samples	Median follow up (months)
Hanif et al. (2020)	2020	USA	1990–2017	85/47	132	58 (21–89)	1–3	Surgery	NA
Dizdar et al. (2017)	2017	Germany	1998–2013	39/38	77	NA	1–3	Surgery	NA
Fotouhi et al. (2016)	2016	Spain	1980–2012	55/57	112	55 (12–91)	1–3	Surgery	12 (1–34)
Cherenfant et al. (2014)	2014	USA	1998–2011	57/71	128	55^ND^ ± 14	1–3	Surgery	33
Ekeblad et al. (2012)	2012	Sweden	1986–2005	50/61	111	53 (21–86)	1–3	Sugery (*n* = 44)	68 (4–416)
Grabowski et al. (2005)	2005	Germany	1981–2001	45/44	89	WDET: 50 (17–84) WDEC: 58.5 (8–84) PDEC: 56 (29–81)	1–3	Surgery + adj. therapy (*n* = 37)	NA

*ND* not defined (median/mean), *NA* not available, *WDET* well differentiated neuroendocrine tumor, *WDEC* well differentiated neuroendocrine carcinoma, *PDEC* poorly differentiated neuroendocrine carcinoma

**Table 2:** Methodological characteristics of included studies

First author	Sample storage	Method	Antibody (dilution)	Cut-off value	Expression (%)	Subcellular localization	Analyzed variables	Outcome	HR estimate	HR	95% CI
Hanif et al. (2020)	FFPE	IHC	Rabbit mAb, clone EP119, BioSB)	> 0% positivity	51.5	Nuc	A, S, Ra, Smo, PS, G, CgA, St, TS, TPH, Ki-67	OS + PFS	UV	OS: 2.89 PFS: 1.55	OS: 1.68–4.95 PFS: 0.93–2.59
Dizdar et al. (2017)	FFPE	IHC	Rabbit pAb, 1:750, NB500-201, Novus Biologicals	IRS > 2 (intensity and percentage)	Cyt: 79.2 Nuc: 40.3	Cyt + Nuc	S, T, N, M, G, R, PS	OS	UV	Nuc: 4.52 Cyt: 29.21	Nuc: 1.53–13.39 Cyt: 0.14-open
Fotouhi et al. (2016)	FFPE	IHC	pAb, Ab469; Abcam	intensity score (nuclear SVV > 1, total SVV > 2)		Cyt + Nuc	NA	OS + PFS	SC MV	OS: Nuc: 1.73 PFS: Nuc: 4.46 OS: Cyt + Nuc: 1.9 PFS: Cyt + Nuc: 6.3	Nuc: 0.76–3.92 Nuc: 2.12–9.39 Cyt + Nuc: 0.3–11.9 Cyt + Nuc: 1.3–30.2
Cherenfant et al. (2014)	FFPE	IHC	Thermo Scientific Lab Vision, 1:50	≥ 50%	Cyt: 76–83 Nuc: 74	Cyt^§^ + Nuc	G, M	OS	MV^$^	Nuc: 3.07 Cyt: 13.07	Nuc: 1.2–18.1 Cyt: 1.3–37.7
Ekeblad et al. (2012)	FFPE	IHC	Mouse mAb, 1:50, sc-17779, Santa Cruz Biotechnology	≥ 5% positivity	Nuc: 27.9 Cyt: 64.9	Cyt + Nuc	G	OS	MV	Nuc: 5.7 Cyt: 0.94	NA
Grabowski et al. (2005)	FFPE	IHC	0.25 µg/ml, Novus Biologicals	> 5% positivity	(Cyt: 41 Nuc: 18 Cyt + Nuc: 4.3)*	Cyt + Nuc	G	OS	SC	Nuc: 4.56^#^	Nuc: 1.66–12.54^#^

*S* sex, *A* age, *N* lymph node metastasis, *M* metastasis, *T* T stage, *G* WHO classification, *Smo* smoking, *PS* primary site, *Ra* race, *TS* tumor size, *St* stage, *TPH* tryptophan hydroxylase expression, *CgA* chromogranin A, *FFPE* formalin-fixed paraffin-embedded, *Nuc* nuclear, *Cyt* cytoplasmic, *SVV* surviving, *IHC* immunohistochemistry, *UV* univariate, *MV* multivariate, *OS* overall survival, *PFS* progression free survival, *RFS* recurrence/relapse free survival, *SC* survival curve, *NA* not available

^*^Results from 139 tumor microsections

^#^Estimated only in WDEC

^§^Clinicopathathological data only for cytoplasmic survivin available

^$^Reported as Odds Ratio

**Table 3:** Study quality assessment according to the ELCWP Scale

	Design	Laboratory methodology	Generalizability	Results analysis	Global score (%)
Hanif et al. (2020)	8	5	7	5	52.08
Dizdar et al. (2017)	8	7	7	7	60.41
Fotouhi et al. (2016)	7	7	4	7	52.08
Cherenfant et al. (2014)	5	5	4	6	41.67
Ekeblad et al. (2012)	7	6	3	5	43.75
Grabowski et al. (2005)	8	7	6	5	54.17

### Study results and meta-analysis

**Fig. 2:**
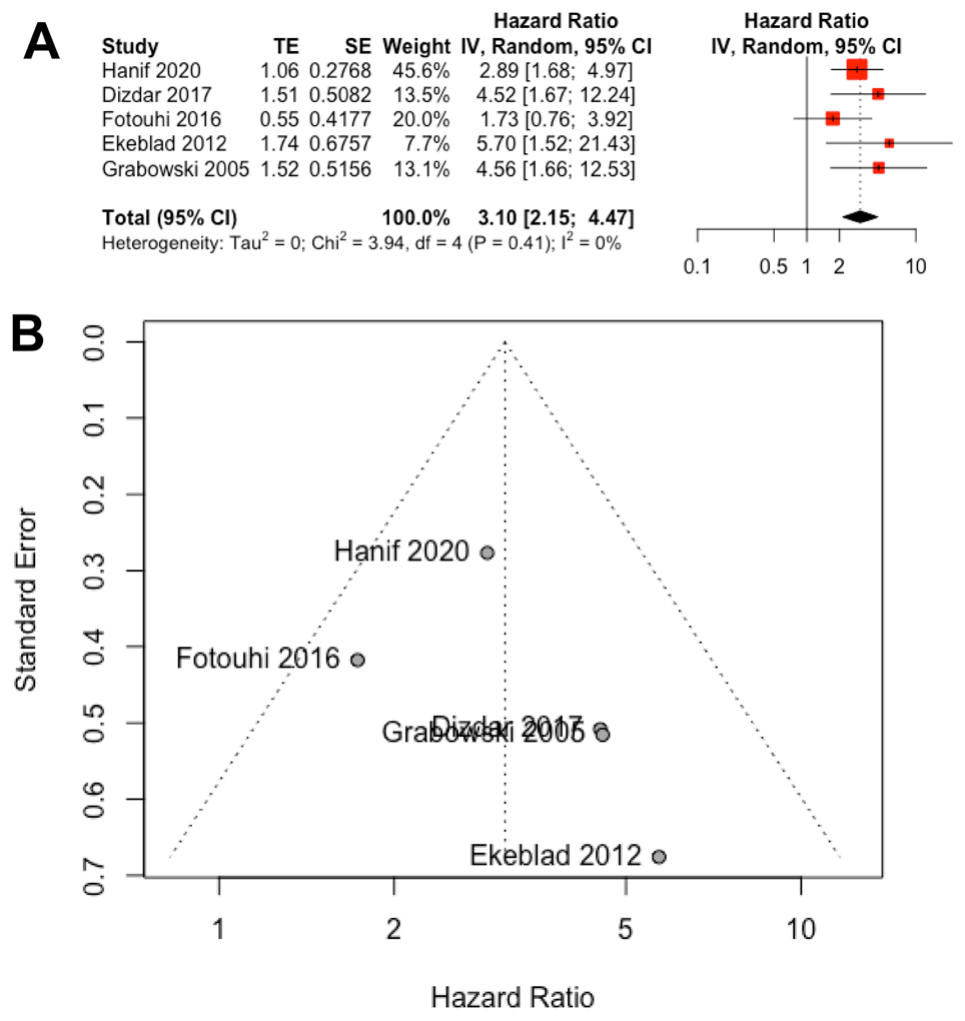
Meta-analysis comparing immunohistochemical expression levels of nuclear survivin with overall survival in GEP-NEN patients. **A** The forest plot depicts the individual and pooled HR with 95% CI. Heterogeneity was estimated by the Chi-squared test and inconsistency statistic (*I*^2^). **B** The funnel plot appears symmetric

First, we were interested in whether survivin is a predictive biomarker for patients suffering from GEP-NEN. Thereby, the majority of studies (*n* = 6) focused on an association between nuclear survivin and overall survival. However, the study published by Cherenfant (Cherenfant et al. 2014) estimated only the mortality by OR and therefore had to be excluded from this analysis. Consequently, we calculated the pooled HR from the remaining 5 studies which demonstrated that high nuclear survivin expression was associated with shorter overall survival (HR 3.10; 95% CI 2.15–4.47; *p *< 0.0001) (Fig. 2A). Note, only 1 study (Ekeblad et al. 2012) provided for nuclear survivin the HR from multivariate analysis and we had to extract survival data from Kaplan–Meier curves of 2 studies (Grabowski et al. 2005; Fotouhi et al. 2016). For the remaining studies, we included HRs from univariate analysis. Furthermore, Grabowski et al. (Grabowski et al. 2005) investigated the expression of nuclear survivin only in the subgroup of G2 tumors. When performing Chi^2^-test (*p *= 0.24) and measuring inconsistency (*I*^2^ = 27%), an important heterogeneity became not evident. Moreover, funnel plot symmetry (Fig. 2B) and performance of Egger’s (*p *= 0.33) and Begg’s (*p *= 0.05) test displayed no risk of publication bias. To further confirm the robustness of these results, we performed one-way sensitivity analysis by alternately removing each study and reassessing the pooled HR of the remaining ones. Importantly, one-way sensitivity analysis confirmed the stability of our findings as the results remained unchanged (data not shown). Even though we only knew the clonality of the antibodies in four publications, we performed a subgroup analysis in this regard. Two studies used a monoclonal (Ekeblad et al. 2012; Hanif et al. 2020) and polyclonal (Fotouhi et al. 2016; Dizdar et al. 2017) antibody, respectively. The HR of the respective subgroups supported that a high expression of survivin was associated with an unfavorable prognosis (monoclonal: HR 3.19; 95% CI 1.93–5.26; *p *< 0.0001; Chi^2^-test: *p *= 0.35; *I*^2^ = 0%; polyclonal: HR 2.68; 95% CI 1.05–6.83; *p *< 0.04; Chi^2^-test: *p *= 0.14; *I*^2^ = 53%). Note that due to the small number of included studies, we were unable to perform a subgroup analysis with regard to the method for HR estimation (univariate versus multivariate). Two studies (Fotouhi et al. 2016; Hanif et al. 2020) investigated the association between expression levels of nuclear survivin and progression-free survival, but did not provide any statistical significance (HR 2.54; 95% CI 0.90–7.13; *p *= 0.0775).

Next, our aim was to determine whether survivin expression is associated with clinicopathological variables in GEP-NEN. In this context, most of the eligible studies focused on an association between survivin and the WHO classification. Since the categories of highly differentiated neuroendocrine tumor and carcinoma (WHO classification 2000) (Solcia et al. 2000) corresponds to the G1/G2 NET of the WHO classification 2010 (Rindi et al. 2010), and the poorly differentiated NEN are defined as NEC (Anlauf 2011), we compared survivin expression between G1/G2 tumors (highly differentiated neuroendocrine tumor and carcinoma) and G3 NEC (poorly differentiated NEN).

**Fig. 3:**
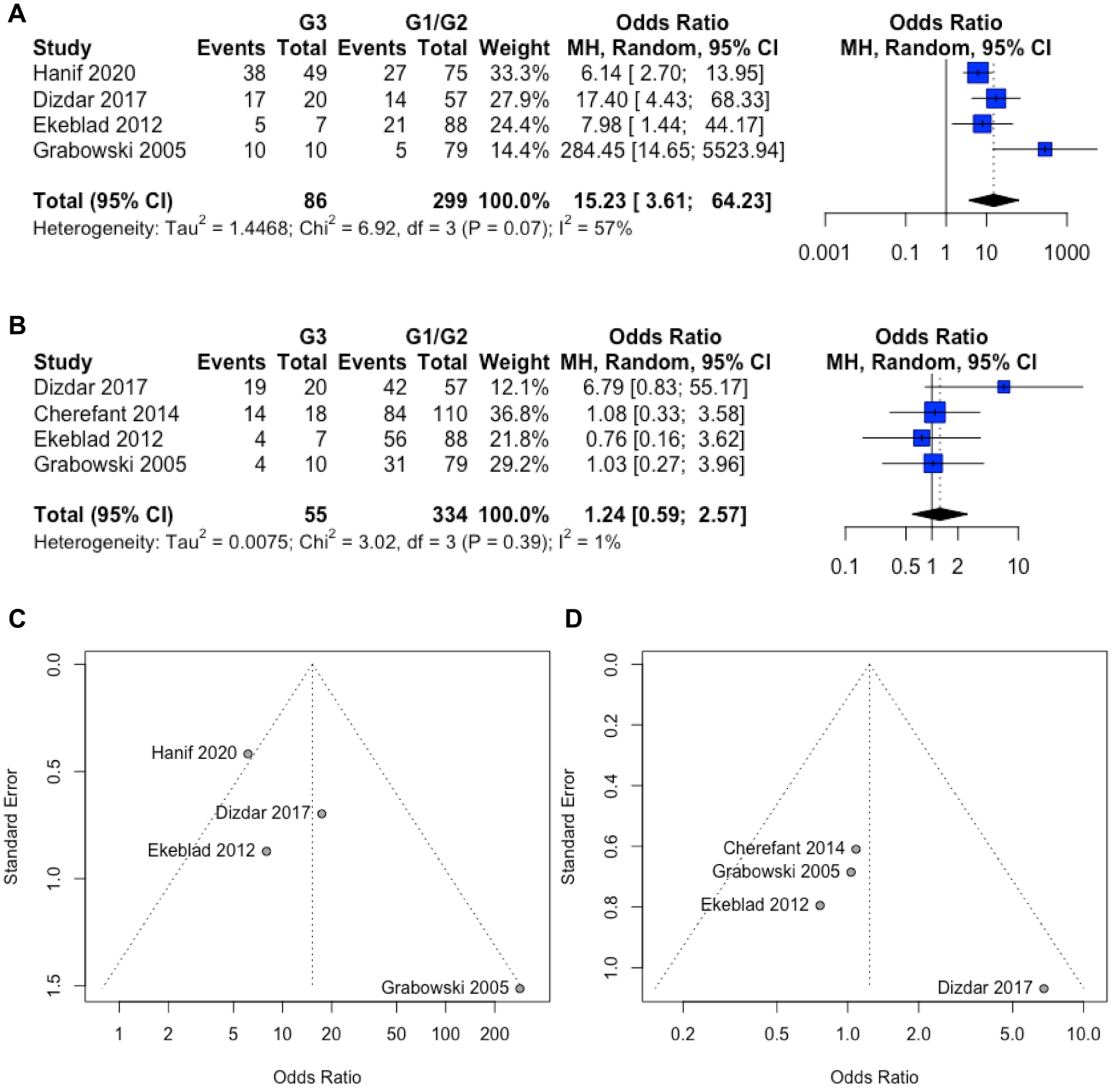
Association between the expression of **A** nuclear and **B** cytoplasmic survivin and grading in GEP-NEN. For each study, the number of GEP-NEN specimen with high or positive expression of survivin (event) and the total number of analyzed samples are outlined. Forest plots show the individual and pooled OR with 95% CI. Chi-squared test and inconsistency (*I*^2^) were applied. Funnel plots for **C** nuclear and **D** cytoplasmic survivin were drawn to visualize publication bias

In contrast to cytoplasmic survivin (OR 1.24; CI 0.59–2.57; *p *= 0.57), high expression levels of nuclear survivin (OR 15.23; CI 3.61–64.23; *p *= 0.0002) were associated with G3/poorly differentiated GEP-NEN (Fig. 3A, B). Because results from the Chi^2^-test (*p *= 0.07) together with inconsistency (*I*^2^ = 57%) made us aware of a heterogeneity, we performed one-way sensitivity analysis. Interestingly, heterogeneity disappeared after excluding the study published by Grabowski et al. (Grabowski et al. 2005). A possible explanation for this observation is that this study found a positive staining of survivin only in 6.3% of the highly differentiated neuroendocrine tumors and carcinomas (G1/G2 NET), while the remaining studies described this in 23.9–36% of G1/G2 GEP-NEN. Furthermore, Funnel plots (Fig. 3C, D) together with Harbord’s (nuclear survivin: *p *= 0.24; cytoplasmic survivin: *p *= 0.57) and Peters’s (nuclear survivin: *p *= 0.50; cytoplasmic surviving: *p *= 0.43) test revealed no publication bias.

Other clinicopathological parameters such as distant metastasis (cytoplasmic survivin: OR 0.51; 95% CI 0.22–1.21; *p *= 0.13) and sex (nuclear survivin: OR 1.23; 95% CI 0.70–2.15; *p *= 0.48) did not provide any evidence of a relationship to survivin expression levels. However, we have to assume that there were only 3 studies (Cherenfant et al. 2014; Dizdar et al. 2017; Hanif et al. 2020) from which we could extract this information. Other parameters such as lymph node metastasis, positivity of resection margins were not associated with expression levels of survivin (Dizdar et al. 2017). Interestingly, Hanif and co-workers as well as our group demonstrated a correlation between survivin and tumor localization (Dizdar et al. 2017; Hanif et al. 2020). In addition, Hanif et al. found higher survivin expression levels in patients with advanced age and larger tumors (Hanif et al. 2020).

## Discussion

In contrast to the increasing incidence of GEP-NEN, the prognosis has improved in recent years due to innovative and interdisciplinary treatment strategies. Especially for unresectable GEP-NEN with low tumor burden and Ki67 proliferation index < 10%, a treatment that targets somatostatin receptors (SSTRs) using somatostatin analogues (SSA) is recommended (Rinke et al. 2009; Caplin et al. 2014). For patients with pancreatic NET and high tumor burden, Ki67 ≥ 10% or progressive disease, a chemotherapy based on a regimen using streptozotocin and temozolomide in combination with 5-fluorouracil or capecitabine are available (Rinke et al. 2021). Targeted therapies including the mTOR inhibitor everolimus and multikinase inhibitor sunitinib were approved for progressive pancreatic NET. In addition, everolimus can be considered for progressive intestinal G1/G2 NET. However, high grade tumors rarely express SSTRs and are therefore unsuitable for a therapy using somatostatin analogues. Thus, for stage IV NEC G3 a chemotherapeutic regimen with cisplatin or carboplatin and etoposide serves as first line therapy (Heetfeld et al. 2015). In contrast, platinum-based concepts seem to be less effective in NET G3 and a standardized therapy has yet to be established (Heetfeld et al. 2015). Albeit overall survival for localized GEP-NEN can be excellent, a survival time of 12 months for patients with distant metastasis (Dasari et al. 2017) underlines the urgent need for new therapeutic concepts to beat this devastating disease.

During the last decades, efforts for the identification of promising tumor-specific markers have been made that allow the development of innovative targeted therapies in GEP-NEN. For example, C-X-C chemokine receptor type 4 (CXCR4) expression increases with G3 tumors (Kaemmerer et al. 2015) and tyrosine kinase receptors c-KIT as well as platelet derived growth factor receptor alpha (PDGFR*α*) were reported to be a negative prognostic marker in pancreatic NET (Knösel et al. 2012). Moreover, programmed cell death ligand (PD-L1) seems to be a prognostic marker in GEP-NEN (Wang et al. 2019) and is now under investigation as therapeutic target in the Phase Ib/II “PLANET” trial (ClinicalTrials.gov Identifier: NCT03043664). Other interesting targets in distinct subset of GEP-NEN include the Ras-Raf-MEK-ERK pathway, heat shock protein (HSP90), Aurora A kinase, focal adhesion kinase (FAK) and histone deacetylases (HDACs) (Aristizabal Prada and Auernhammer 2018; Dizdar et al. 2019).

In this context, IAP family member survivin could also be of interest as a therapeutic target in GEP-NEN. Because survivin is highly expressed in a large number of tumors and nearly undetectable in normal adult tissues, it has become an attractive molecule for novel cancer therapies. Indeed, a number of clinical trials that evaluated the efficacy of small molecule survivin antagonist YM155 alone or in combination with chemotherapeutic agents in distinct tumors have been completed (Tolcher et al. 2008, 2012; Giaccone et al. 2009; Satoh et al. 2009; Lewis et al. 2011; Kelly et al. 2013; Clemens et al. 2015; Kudchadkar et al. 2015; Papadopoulos et al. 2016; Shimizu et al. 2020). Recently, we demonstrated in cell lines originating from NECs of the gastroesophageal junction and colon a marked pro-apoptotic effect of YM155 in vitro (Dizdar et al. 2017). In addition, YM155 as well as transduction of these cell lines with survivin specific shRNAs inhibited tumor growth in our xenograft mouse model. Although results from first clinical Phase I and II trials demonstrated rather frustrating results even in combination with chemotherapeutics, achieving maximally partial response rates, survivin seems to remain an attractive target for cancer treatment (Tolcher et al. 2008, 2012; Giaccone et al. 2009; Satoh et al. 2009; Lewis et al. 2011; Kelly et al. 2013; Clemens et al. 2015; Kudchadkar et al. 2015; Papadopoulos et al. 2016; Shimizu et al. 2020). For example, terameprocol (EM1421, M4N), that disrupts the activity of transcription factor Sp1 and thereby prevents transcription of survivin, also inhibited cell proliferation of our GEP-NEC cell lines (Dizdar et al. 2017) and has been under consideration in a phase I clinical trial including patients with solid tumors (ClinicalTrials.gov Identifier: NCT00259818). In this context, it must also be emphasized that one reason for the observed minimal response to YM155 could be the chemical instability of YM155, which leads to a significant reduction in antitumor efficacy (Li et al. 2019). Thus, pharmacokinetic studies demonstrated that after YM155 treatment, a rapid decrease in the concentration of YM155 in serum and tumor was observed (Nakahara et al. 2007). Another reason could be that formal pharmacokinetic interaction analyses between survivin and chemotherapeutic agents in combination treatments have not been investigated (Clemens et al. 2015). In addition, the response rate to YM155 targeted therapy may be dependent on the level of survivin expression in the tumors being treated, but this has generally not been analyzed in phase I/II trials to date.

Beyond a small molecule approach, there is an attempt to improve survival through immunotherapy based on vaccines. Consequently, it is not surprising that a phase I study is currently recruiting patients with metastasized pancreatic NET, in which a survivin long peptide vaccine is under investigation (ClinicalTrials.gov Identifier: NCT03879694).

An essential prerequisite for a targeted therapy, however, is the expression of the target structure within the tumor to be combated. Thus, we conducted a systematic review and meta-analysis to elucidate the role of survivin as biomarker in GEP-NEN. Using our pre-defined search criteria, we identified 6 studies that analyzed the expression of survivin in GEP-NEN (Grabowski et al. 2005; Ekeblad et al. 2012; Cherenfant et al. 2014; Fotouhi et al. 2016; Dizdar et al. 2017; Hanif et al. 2020). All of these studies used immunohistochemistry to detect survivin and described mostly both, nuclear and cytoplasmic expression patterns in tumor cells. Our meta-analysis revealed a significant association between high nuclear survivin and overall survival. Although heterogeneity became not evident, we have to draw attention to the fact that only a single study provided data from multivariate analysis (Ekeblad et al. 2012) and another one included only well differentiated endocrine carcinomas into their survival analysis (Grabowski et al. 2005). In some cases, time-to-event data were reconstructed from the original publication to obtain HR etsimates and might be therefore less accurate.

The second important observation of our meta-analysis was that highly proliferative G3 NEC showed increased expression levels of nuclear survivin compared to G1/G2 tumors, which fits perfectly with the fact that nuclear survivin acts as a regulator of cell division. However, we have to admit that the WHO classification of GEP-NEN changed over the past decades and not all studies applied the proliferation-based classification system from 2010. On the other hand, categories “well differentiated neuroendocrine tumor” and “well differentiated neuroendocrine carcinoma”, as defined in the WHO classification from 2000, correspond to the G1/G2 NET in the classification from 2010 and poorly differentiated neuroendocrine carcinoma correspond to NEC G3 (Anlauf 2011).

Importantly, we must also emphasize that our meta-analysis has some limitations attributed to the methodological variability of the included studies. Most of them performed immunohistochemistry using different antibodies, quantification methods and cut-off values to define positivity. Moreover, some studies included only patients with pancreatic NET, one study involved also patients with neuroendocrine tumors of the lung.

Furthermore, we identified only a small number of published retrospective studies that were available from standard research databases and did not include grey literature. Because positive results are more likely to be published, we may have therefore introduced a publication bias. Even though we performed statistical analysis to test the likelihood of publication bias, the informative value of these tests should be treated with caution due to the small number of studies.

## Conclusion

Although only a small number of eligible studies could be included in our meta-analysis, the results are consistent with meta-analyses supporting survivin as prognosticator in other tumor entities (Krieg et al. 2013a, b; Fung et al. 2021). Interestingly, nuclear survivn did not only correlate with a poor outcome, but also with a more aggressive tumor grading in GEP-NEN. Future studies of larger patient cohorts including a training and validation set, using a standardized and validated immunohistochemical staining method, will be needed to confirm these observations.

## Data Availability

The datasets used and/or analyzed during the current study are available from the corresponding author on reasonable request.
